# New strategies to improve communication in the intensive care unit during the COVID-19 pandemic

**DOI:** 10.1186/s13054-022-04057-2

**Published:** 2022-06-28

**Authors:** Charlene Kit Zhen Chua

**Affiliations:** 1 Department of Intensive Care and Hyperbaric Medicine, Alfred Health, Melbourne, VIC Australia; 2 School of Medicine and Public Health, University of Newcastle, Newcastle, NSW Australia

**Keywords:** Communication, COVID-19, Family liaison service, Hands-free communication device, Intensive care, Role labels, Videoconferencing

## Introduction

Communication with patients’ family members in the intensive care unit (ICU) has been extremely challenging during the COVID-19 pandemic. Strict visitation policies present a significant barrier for healthcare practitioners to provide medical information, offer emotional support and build rapport with patients’ family members. As a result of being unable to see the patients at the bedside, family members can feel frustrated, depressed and anxious, affecting their own emotional and psychological well-being [1]. Two strategies that can be employed for effective communication with patients’ family members include the establishment of an ICU family liaison service and the use of videoconferencing.

The COVID-19 pandemic continues to take a huge toll on interpersonal communication among healthcare staff in the ICU. The application of face masks [2] and other personal protective equipment (PPE) [3], social distancing, work isolation and redeployment of staff can change the dynamics of team communication. Non-verbal communication methods such as hand signals [4] and the use of identification adjuncts to improve staff identification [5] have emerged as methods to address these challenges. In addition, the effect of these disruptions can be mitigated by the application of hands-free communication devices as well as the introduction of team roles and name labels.

## Strategies

### ICU family liaison service

The ICU family liaison service is a new initiative started during the pandemic to ensure timely and effective communication with patients’ family members. The family liaison team is responsible for contacting every patient’s family within 24 h of ICU admission. The team notifies the family about the patient’s ICU admission, addresses immediate concerns, clarifies details of next-of-kin, offers information about the visitation process (including applicable restrictions) and provides contact details of the unit.

The team maintains contact and supports the family throughout the admission. Any concerns are escalated appropriately to specific services such as medical, allied health or social support departments. The sole responsibility in providing updates about a patient’s clinical progress and management plan lies with the medical staff. This is important to prevent miscommunication as the family liaison team is not involved in medical management of the patient.

The ICU family liaison service assists the medical team by ensuring the patients’ family members have convenient access to information regarding the ICU admission. The introduction of the family liaison team has been shown to reduce pressure on medical staff [6] and improve levels of satisfaction for patients’ family members [7].

### Videoconferencing

Web-based videoconferencing has become a routine communication channel during the pandemic, particularly when family visits have not been allowed or were impractical (for example due to government-imposed ‘lockdowns’). Videoconferencing offers a remote virtual meeting opportunity for ‘real-time’ discussion between healthcare staff and patients’ family members [8]. It is the most practical alternative option to an in-person meeting or a phone call, especially when breaking bad news [9], delivering complex medical information or discussing goals of care.

Conscious and lucid patients in the ICU are encouraged to use videoconferencing to connect with their families to promote their emotional and psychological well-being. Other benefits of videoconferencing include preventing delirium, improving engagement with health services, providing mental support, enhancing staff morale and overcoming language barriers [10].

An initial nurse-led approach to facilitate a virtual walk-around of the patient’s bed space helps with the family’s understanding of the patient’s clinical status. This is followed by clinical updates from ICU medical staff. Specific concerns and questions can be further addressed at the end of the virtual visit. The timing and duration of the virtual visit are flexible. This practice provides reassurance, builds rapport and helps generate trust with the patient’s family.

### Hands-free communication devices

The use of small, wearable, voice-activated, hands-free communication devices allows healthcare staff to connect and communicate instantly with their colleagues by identifying their names or roles. It helps to decrease the risk of infection by reducing the use of individual mobile phones and exposure to personal clothing [8]. It also saves time and PPE resources as direct assistance and support can be sought immediately without leaving the bed space. Hands-free conversations can be initiated and continued with no interruption to clinical tasks, improving work efficiency. The staff has control over its practical use by declining incoming calls when clinically inappropriate [11]. The correct use of hands-free communication devices has allowed staff to feel supported and stay connected despite working in a difficult and potentially isolating environment during the pandemic.

### Team role and name labels

The use of PPE can impact communication and interaction among healthcare staff [12] and lead to difficulties recognising and identifying other staff [5]. Important aspects of communication such as facial expressions, clarity of voice and volume of speech are also negatively affected by PPE. The application of colour-coded team roles and name labels is a useful method to improve communication in this setting. The identification labels are usually attached over the hospital gowns at the donning station supervised by a ‘PPE monitor’. Names can be written over the top part of face shields. This method can be effective in a large intensive care unit, especially with staff redeployment and new staff recruitment. The use of labels as visual aids allows staff to recognise each other in a new working environment, promoting the efficient transfer of information and improving team dynamics. In resuscitation situations, it enables immediate identification of staff members for team role allocation.

## Conclusion

ICU staff has had to innovate and develop new communication strategies to address the barriers brought about by the COVID-19 pandemic. This has resulted in a paradigm shift from the traditional communication methods used pre-pandemic. These strategies may be adapted in other intensive care units to improve patient families’ access to information and enhance staff communication and teamwork. Future studies should focus on refining, diversifying and innovating elements of formal and informal communication within the ICU considering workforce and workplace challenges due to COVID-19.

## Data Availability

Not applicable.

## Abbreviations

ICU: Intensive care unit
PPE: Personal protective equipment
